# Hyperparameter optimization ResNet by improved Beluga Whale Optimization

**DOI:** 10.1371/journal.pone.0333575

**Published:** 2025-10-24

**Authors:** Huan Liu, Shizheng Qu, Shuai Zhang, Yingxin Zhang, Yanqiu Li

**Affiliations:** 1 School of Data Science and Artificial Intelligence, Jilin Engineering Normal University, Changchun, China; 2 School of Computer Science and Technology, Changchun University of Science and Technology, Changchun, China; 3 Faculty of Mathematics, University of Waterloo, Waterloo, Canada; Dayananda Sagar University, INDIA

## Abstract

The parameter values of neural networks will directly affect the performance of the network, so it is very important to choose the appropriate parameter tuning method to improve the performance of the neural network. In this paper, the improved beluga whale optimization hyperparameter optimization ResNet model is used to construct a new model, EBWO-ResNet. Firstly, in order to solve the problem that the initial population of the original beluga whale optimization is not rich enough, the Tent chaotic map is introduced into the beluga whale optimization, and a new algorithm EBWO is constructed. Secondly, in order to solve the problems of low accuracy and difficult parameter tuning of ResNet, the EBWO algorithm was integrated into ResNet to construct a new model EBWO-ResNet. Finally, in order to verify the effectiveness of the EBWO algorithm, the EBWO algorithm was applied to three engineering problems and compared with other five swarm intelligent algorithms, and in order to verify the effectiveness of the EBWO-ResNet model, EBWO-ResNet was applied to maize disease identification,in order to improve the accuracy of corn identification and ensure corn yield,and the other seven models were compared based on three evaluation indexes. The experimental results show that the EBWO algorithm provides the best solutions in the three engineering problems, and the EBWO-ResNet has the best performance in identifying maize diseases, with an accuracy of 96.3%,which is 0.2-1.5 percentage points higher than that of other models.

## Introduction

Neural networks have attracted attention since they were proposed, especially for datasets with a large number of samples, and neural networks can be trained and fitted by a large number of samples. The neural network has an embedded feature extraction structure, so the neural network can perform feature extraction autonomously without manual operation, which can improve the quality of feature extraction and help for subsequent work. Based on the above advantages, neural networks are trusted and widely used in medicine [1], agriculture [2], fault diagnosis [3], and other fields. However, with the increase of social demand, the disadvantages of traditional neural networks have gradually begun to appear, and people’s requirements for their performance have gradually begun to increase. Based on this, a large number of studies have begun to focus on neural network optimization, and a large number of neural network variants have begun to appear.

Wang et al. designed an HCNN based on global inference [4], in which only one layer of a three-dimensional convolutional neural network and a one-layer two-dimensional convolutional optical network were used to jointly extract signs, and SS-GloRe-Unit was also designed to completely extract global features. In order to improve the performance of ResNet, Yang et al. proposed a MA-ResNet model based on multiple attention mechanisms [5], and the experimental results show that the proposed model has faster convergence speed, higher accuracy, and better accuracy of small target classification than other feature extraction models. Yang et al. proposed an rE-GoogLeNet convolutional neural network model [6],which replaced the convolutional kernel of the first layer, added the ECA attention mechanism to the Inception module, used the improved module of residual network connection, replaced the ReLU activation function with the leaky ReLU activation function, and finally simplified the auxiliary classifier, and the simulation results show that the proposed model has the best performance. It can be seen that the optimization of the neural network can actually improve the performance of the model, especially the internal parameter values of the neural network can directly affect the performance of the model, and it is of great significance to select the appropriate parameter values to improve the performance of the model. Traditional neural network parameter tuning mainly relies on manual, grid search and random search, which is inefficient and time-consuming, especially when it is difficult to find the optimal parameter combination for manual parameter tuning.

In recent years, meta-heuristic algorithms have been applied to solve complex optimization problems due to their powerful optimization ability, and good results have been obtained. When it is applied to solve the problem of neural network parameter optimization, it also shows powerful optimization ability. Guo et al. first proposed the dung beetle optimized convolutional neural network (DBO-CNN) [7], in which the dung beetle optimization algorithm hyperparameters were used to optimize the convolutional neural network, and the experimental results proved that the accuracy of DBO-CNN reached 97.93%. Wang et al. proposed an Adaptive Gaussian Variation PSO (ADGMPSO) algorithm [8] and applied it to the parameter configuration of the optimized convolutional neural network, and the experimental results show that the optimized neural network has higher accuracy and generalization ability. In order to improve the performance of CNN, Gadekallu et al. optimized the hyperparameters of CNN by applying the newly developed meta-heuristic algorithm HHO, and the proposed hybrid model of HHO-CNN [9] performed better than the existing model through comparative analysis. Heng et al. proposed a new hybrid neural network model based on algorithm optimization [10], in which the long short-term memory (LSTM) network is used to capture the time series features and depth features of CNN output, and the slime bacteria algorithm (SMA) algorithm is used to adaptively configure the hyperparameters of the LSTM in order to optimize the hyperparameter configuration of the LSTM.

In summary, it is feasible to apply meta-heuristics to replace the traditional manual parameter tuning method of neural network. In this paper, the Beluga optimization algorithm is improved by using the Tent chaotic map, and the improved Beluga optimization algorithm is integrated into the ResNet model to achieve the purpose of adaptively modifying the parameters. The rest of this article reads as follows:

The second part details the methods proposed in this paper. The third section describes the datasets, experiment configurations, and experimental results used for experiments. The fourth part summarizes the experiment and clarifies the future research directions.

## Experimental methods

### Improved Beluga Whale Optimization

#### Beluga Whale Optimization.

The Beluga Whale Optimization [11] [12] [13] is a new meta-heuristic algorithm developed in 2022, which simulates the life behavior of beluga whales in nature, just like other meta-heuristic algorithms that simulate population behavior in nature. Beluga whales are social animals, and they move together, exchange information, and hunt and breed. Beluga whales have natural predators and can also have accidents, so beluga whales can also die, a phenomenon called "whale fall". The beluga whale optimization algorithm is divided into three stages in mathematical modeling, which are swimming, predation, and whale fall. In the Beluga optimization algorithm, each individual is treated as a separate search agent, and the initial search agent matrix is modeled as follows:

$$X=\left[\begin{array}{cccc} x_{1}^{1} & x_{1}^{2} & \ldots & x_{1}^{dim}\\ x_{2}^{1} & x_{2}^{2} & \ldots & x_{2}^{dim}\\ \vdots & \vdots & \ddots & \vdots \\ x_{n}^{1} & x_{n}^{2} & \ldots & x_{n}^{dim} \end{array}\right]$$ (1)

In the exploration phase of the beluga whale optimization algorithm, the swimming behavior of the beluga whale is mathematically modeled, and the calculation formula is as follows:

$$\left\{ \begin{array}{c} X_{i,j}^{t+1}=X_{i,d_{j}}^{t}+\left(X_{r,d_{1}}^{t}-X_{i,d_{j}}^{t}\right)\left(1+r_{1}\right)\sin\left(2\pi r_{2}\right),j=\text{ even }\\ X_{i,j}^{t+1}=X_{i,d_{j}}^{t}+\left(X_{r,d_{1}}^{t}-X_{i,d_{j}}^{t}\right)\left(1+r_{1}\right)\cos\left(2\pi r_{2}\right),j=\text{ odd } \end{array}\right.$$ (2)

Where *t* represents the current iteration, $X_{i,j}^{t+1}$ represents the position information of the *i* individual in the j dimension, *d*_*j*_ is randomly selected in the dimension, $(X_{r,d_{1}}^{t}$ represents the position of the *r* individual, and *r* represents random selection. *r*_1_ and *r*_2_ represent random numbers with values in the range of (0,1).

In the development stage of the BWO, the predatory behavior of beluga whales was mathematically modeled by simulating the predatory behavior of beluga whales, and the calculation formula is as follows:

$$X_{i}^{t+1}=r_{3}X_{B}^{t}-r_{4}X_{i}^{t}+C_{1}\times \mathrm{Levy}\times \left(X_{r}^{t}-X_{i}^{t}\right)$$ (3)

$X_{B}^{t}$ represents the best position in the individual, *r*_3_ and *r*_4_ like *r*_1_ and *r*_2_, represents a random number with a value range of (0,1). The formula for calculating *C*_1_ is as follows:

$$C_{1}=2r_{4}\left(1-\frac{t}{T}\right)$$ (4)

where *T* represents the maximum number of iterations. The formula for calculating the *Levy* function is as follows:

$$\text{ Levy }=0.05\times \frac{u\times \sigma }{|v{|}^{\frac{1}{\beta }}}$$ (5)

$$\sigma ={\left(\frac{\Gamma (1+\beta )\times \sin(\pi \beta /2)}{\Gamma \left((1+\beta )/2)\times \beta \times 2^{(\beta -1)/2}\right)}\right)}^{1/\beta }$$ (6)

Where *u* and *v* are random numbers that obey a normal distribution, and *β* is the default constant.

There is a balance factor *B*_*f*_ in the Beluga optimization algorithm to determine whether the Beluga optimization algorithm will shift from exploration to development, and the calculation formula is as follows:

$$B_{f}=B_{0}\times \left(1-\frac{t}{2T}\right)$$ (7)

where *B*_0_ varies randomly between (0,1).

The Beluga optimization algorithm sets the beluga fall step parameter *X*_*s*_ when simulating whale falls for mathematical modeling, and the calculation formula is as follows:

$$X_{s}=(UB-LB)\exp\left(-C_{2}\frac{t}{T}\right)$$ (8)

where *UB* and *LB* are the upper and lower limits of the variable, respectively, and $C_{2}=2W_{f}\times n$ is the step factor, *W*_*f*_ is calculated as follows:

$$W_{f}=0.1-\frac{0.05t}{T}$$ (9)

Therefore, the mathematical modeling formula of the beluga optimization algorithm for simulating whale falls is as follows:

$$X_{i}^{t+1}=r_{5}X_{i}^{t}-r_{6}X_{r}^{t}+r_{7}X_{s}$$ (10)

where *r*_5_, *r*_6_, *r*_7_ are also random numbers with a value of (0,1).

#### The algorithm proposed in this paper.

In order to solve the problem that the initial solution randomness is too large and the population is not abundant enough [14] [15], the Tent chaotic map [16] [17] [18] is introduced into the Beluga optimization algorithm,Tent mapping is a discrete mapping with a simple algorithm but complex sequence, which has the advantages of fast operation speed and uniform sequence distribution by generating pseudo random sequences,and the calculation formula is as follows:

$$x_{n+1}=\left\{ \begin{array}{c} \frac{x_{n}}{\alpha },x_{n}\in [0,\alpha )\\ \frac{\left(1-x_{n}\right)}{1-\alpha },x_{n}\in [\alpha ,1] \end{array}\right.$$ (11)

The introduction of Tent chaotic mapping into the beluga optimization algorithm can avoid the problem of excessive randomness of the population in the initialization process, because the Tent chaotic map can replace the mechanism of random scattering of the population, so that the population is relatively evenly distributed. The improved algorithm is named EBWO.

### Improve the neural network model

#### Basic ResNet model.

**Fig 1:**
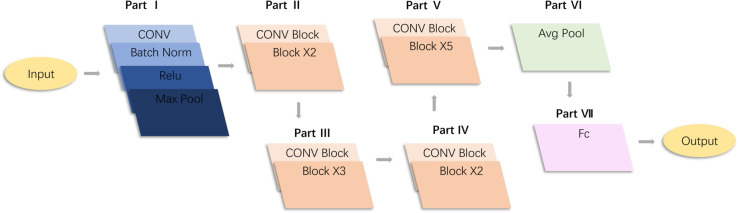
Structure diagram of ResNet50.

Deepening the number of neural network layers can actually improve the accuracy of the neural network [19] [20] [21], but after the threshold is reached, the accuracy of the neural network will drop significantly, which is obviously contrary to the conclusion that "the more layers of the network, the higher the network accuracy", which researchers call a degenerative phenomenon. The degradation phenomenon makes the neural network unable to do linear transformation well, which leads to problems such as gradient disappearance and gradient explosion. To address these issues, ResNet [22] [23] [24] introduces shortcut links that allow the output of one layer to skip one or more layers directly and connect to the input of subsequent layers. This makes it possible for certain network layers to pass information even without any transformation. In this experiment, ResNet50 was selected as the basic model, and the structure of ResNet50 is shown in Fig 1.

#### The model proposed in this paper.

In this paper, the traditional parameter tuning method is abandoned, and the improved EBWO algorithm is used to adaptively find the best parameter combination of ResNet,parameters including training algorithm, momentum leaning, batch size, epoch, and validation frequency. and the model is named EBWO-ResNet. The EBWO-ResNet pseudocode is as follows:

1. Set the parameters *N* and *T*

2. Initialize the population and calculate the fitness

3. While t≤T

4. For i=1 to N

5. If *B*_*f*_>0.5

6. Update the position according to Eq (2).

7. Else if $B_{f}\leq 0.5$

8. Update the position according to Eq (3).

9. End if

10. End for

11. For i=1 to N

12. If $B_{f}\leq W_{f}$

13. Update the position according to Eq (10).

14. End if

15. End for

16. *t* = *t* + 1

17. End while

18. Output the optimal solution

19. Assign the optimal solution to ResNet50

**Fig 2:**
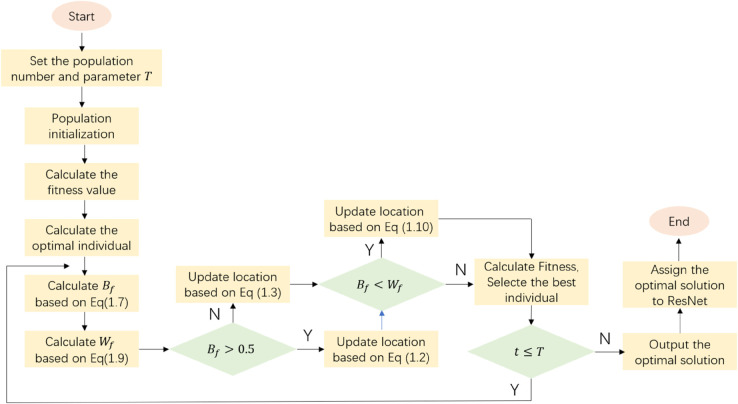
Flow chart of EBWO-ResNet.

The improved EBWO-ResNet model can be adaptively found by the EBWO algorithm to find the optimal solution combination, and directly transmitted back to the ResNet model without manual assignment, as shown in Fig 2.

## Experimental results

### Experimental setup

All the research in this paper was carried out in the laboratory, and the experiments were carried out by MATLAB.The CPU model is AMD Ryzen 7 5800H with Radeon Graphics, the GPU model is RTX 4060, and the OS model is Window 11. In order to verify the effectiveness of the proposed algorithm EBWO, EBWO was applied to three engineering experimental optimization problems, and compared with five other meta–heuristic algorithms, namely BWO, Sine Cosine Algorithm(*SCA*) [25], Grey Wolf Optimizer (GWO) [26], Genetic Algorithm (GA) [27], and Particle Swarm Optimization (PSO) [28].The experiment was run 30 times independently, and the results were averaged. In order to verify the effectiveness of the proposed model EBWO-ResNet, EBWO-ResNet was applied to the identification of maize diseases, and compared with seven ResNet models optimized by other meta-heuristic algorithms, the seven meta-heuristic algorithms were BWO, Fruit Fly Optimization Algorithm (FOA) [29], GWO,PSO, Firefly Algorithm (FA) [30], Ant Colony Optimization(ACO) [31],GA were evaluated as accuracy, sensitivity, and precision.

### Algorithm comparison experiments

#### Pressure vessel design.

One of the many engineering problems is pressure vessel design. In real life, we will inevitably apply it to containers, such as gas storage tanks, oil tanks, and even containers used in the chemical industry to load products. The safety of these containers will directly affect the safety of the product, and the sealing, pressure resistance and other attributes of the container will also affect the use of the product, so it is very important to do a good job in the design of the container to ensure the safe use of the product. There are many problems in the design of containers, such as the thickness, size and structure of containers. In this experiment, four aspects were considered, namely the thickness TS of the container wall, the thickness TH of the hemispherical head, the inner radius R and the length L of the cylindrical cross-section, and the experimental results are shown in Table 1.

**Table 1:** Experimental results of pressure vessel design.

Name of the algorithm	Fitness value	Ts	Th	R	L
EBWO	8792.7454	1.30382	0.671971	67.391	32.8523
BWO	9894.9873	1.30344	0.645015	67.3869	10.0021
SCA	10510.7979	1.30212	0.670182	67.3868	29.1525
GWO	11327.1572	1.3176	0.643469	67.3895	10
GA	12209.2577	1.36381	0.645521	67.3897	10
PSO	16398.3329	1.35945	0.643448	67.3942	10.0012

The smaller the fitness value in the table, the better the optimization performance of the algorithm. As can be seen from Table 1, EBWOA ranks first with the smallest fitness value, and BWO ranks second, with a fitness value of 1102.242 higher than EBWO, indicating that there is still a gap between the optimization performance of the two. In third place is SCA, which has a fitness value similar to BWO, but is very different from EBWO, indicating that SCA’s performance is not as good as EBWO. GWO, GA and PSO ranked in the last three places, and their optimization performance was average, especially PSO, which had the lowest fitness value, with a difference of 7605.585 from EBWO.

#### Three-bar truss design.

The three-bar truss is one of the most common structures in engineering construction, and its main function is to support, so only the three-bar truss has sufficient bearing capacity to ensure the safety of the project. The three-bar truss is mainly composed of three rods, so the bearing capacity of the three-bar truss is mainly related to the material of the rod, the cross-sectional size of different rods, and the connection method. Based on this, this experiment was optimized from the cross-sectional area, and the experimental results are shown in Table 2. Where X1 and X2 represent different cross-sectional areas. Table 2 explains the same as Table 1, and it can be seen from Table 2 that EBWO also provides the lowest fitness value, ranking first, with a fitness value of 263.9106, and shows high stability in the optimization of variables X1 and X2.

**Table 2:** Experimental results of three-bar truss design.

Name of the algorithm	Fitness value	X1	X2
EBWO	263.9106	0.79148	0.40046
BWO	263.9324	0.78171	0.42831
GWO	263.9755	0.77845	0.43796
SCA	264.2026	0.76896	0.46709
GA	264.4968	0.81872	0.32927
PSO	264.7459	0.75661	0.50745

#### Tension and compression spring design.

The tension and compression spring is also one of the most commonly used components in engineering construction, and its main function is to ensure the stability and balance of other machinery, and to ensure the normal operation of the entire mechanical system. In order to achieve this function, the tension and compression spring must have sufficient tension, and the tension of the tension and compression spring is related to the influencing factors such as the characteristics of the material, size, and stiffness coefficient. Based on this, this experiment was optimized from three influencing factors, namely the diameter of the spring coil d, the diameter of the spring coil D, and the number of coils p, and the experimental results are shown in Table 3. From Table 3, it can be seen that the fitness value of EBWO (0.012708) is also better than that of other algorithms, and it shows strong comprehensive performance in the optimization of variables X1, X2 and X3.

**Table 3:** Experimental results of tension and compression spring design.

Name of the algorithm	Fitness value	X1	X2	X3
EBWO	0.012708	0.054343	0.42391	8.3192
BWO	0.012901	0.063716	0.72094	3.1798
SCA	0.013401	0.05	0.31717	14.0697
GWO	0.014855	0.05	0.317251	14.0813
PSO	0.016287	0.054526	0.42868	8.0559
GA	0.017348	0.060659	0.61329	4.2167

### Model comparison experiments

#### Datasets.

**Fig 3:**
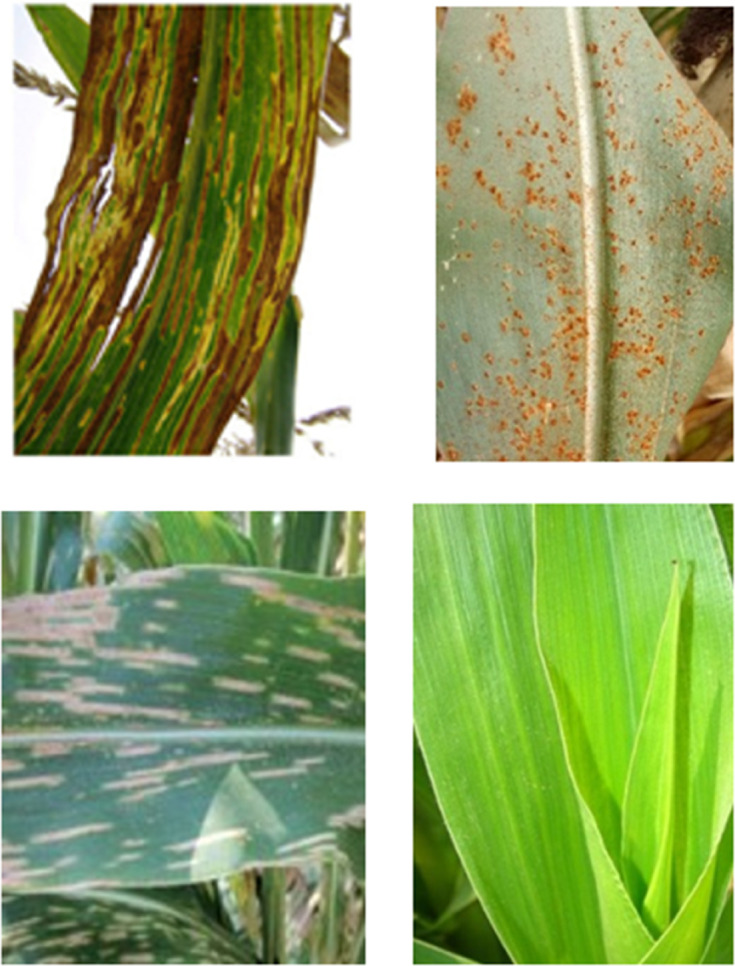
Dataset example.

The dataset is a public dataset derived from the Kaggle open source platform. This experimental dataset contains a total of 4187 images in 4 categories [32], namely blight, common rust, gray leaf spot, and healthy. An example of the dataset is shown in Fig 3.The dataset is divided into training set and test set, of which the training set accounts for 70% and the test set accounts for 30%.

#### Comparison results.

**Fig 4:**
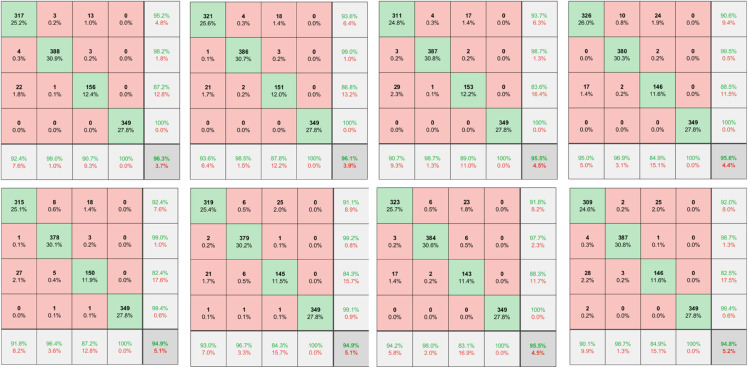
Comparison of models.

The results of ResNet were optimized by applying the different group intelligence algorithms as shown in Fig 4. In order: EBWO-ResNet, BWO-ResNet, FOA-ResNet, GWO-ResNet, PSO-ResNet, FA-ResNet, ACO-ResNet, GA-ResNet. The confusion matrix in Fig 4 consists of 5 rows and 5 columns, of which the first four rows represent the prediction categories, namely blight, common rust, gray leaf spot, and healthy, and the last row represents the sensitivity of model recognition. The first four columns represent the real categories, namely blight, common rust, gray leaf spot, and healthy, and the last column represents the model recognition accuracy. EBWO-ResNet has the highest accuracy rate of 96.3%, BWO-ResNet ranks second, reaching 96.1, a difference of 0.2 percentage points from EBWO-ResNet, among all models, only EBWO-ResNet and BWO-ResNet have an accuracy rate of more than 96%, and the accuracy of other models is below 96%. Among them, GWO-ResNet ranked third, reaching 95.6%, 0.7 percentage points lower than EBWO-ResNet. The accuracy of FOA-ResNet is equal to that of ACO-ResNet, reaching 95.5%. The accuracy of the other models was below 95%, with the same accuracy of 94.9% for PSO-ResNet and FA-ResNet, and the lowest accuracy for GA-ResNet (94.82%), a difference of 1.48 percentage points from EBWO-ResNet. Therefore, the performance of EBWO-ResNet is undoubtedly the best in terms of accuracy.

**Fig 5:**
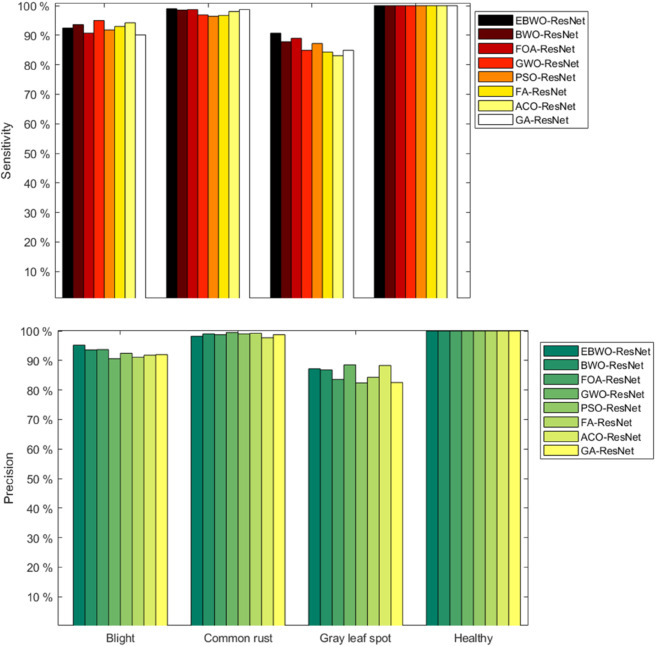
Sensitivity and precision result plots.

Sensitivity and precision results are shown in Fig 5. In order: Sensitivity result plots, precision result plots.For the sensitivity evaluation index, all models achieved 100% in the recognition of healthy, and although EBWO-ResNet did not achieve the best effect in the recognition of blight, EBWO-ResNet achieved the best effect in the recognition of common rust, reaching 99.0%. Most importantly, among all the models, only EBWO-ResNet achieved a recognition performance of more than 90% for all categories, and the other models achieved a recognition performance of more than 90% for gray leaf spots. So on the whole, EBWO-ResNet identification sensitivity is also the best. For the precision evaluation index, only PSO-ResNet, FA-ResNet and GA-ResNet did not reach 100% in identifying healthy, and the other models all reached 100%. And for the precision, no model achieved more than 90% in identifying all categories, EBWO-ResNet provided the best accuracy in identifying blight, reaching 95.2, and the other models only reached 93.7, a difference of 1.5 percentage points from EBWO-ResNet. In the other two categories, EBWO-ResNet also reached 98.2% and 87.2%, respectively, and the results were also satisfactory. Based on the three evaluation indicators, EBWO-ResNet has the best comprehensive performance.

## Conclusion

In order to solve the problem of low accuracy of maize disease identification, an EBWO-ResNet model was proposed. In order to improve the performance of the swarm intelligence algorithm, the Tent map is selected as the improved method, which is integrated into the BWO algorithm, and finally the improved EBWO algorithm is applied to improve the ResNet model. In order to verify the effectiveness of the proposed EBWO algorithm and apply it to engineering experiments, it can be found that the performance of the EBWO algorithm is the best and the most stable, and the best results have been achieved in the three engineering experiments. In order to verify the effectiveness of the proposed model EBWO-ResNet, EBWO-ResNet was applied to maize disease identification, and compared with other 7 population intelligence algorithms, it can be seen from the experimental results that the accuracy of EBWO-ResNet is the highest, reaching 96.3%. In summary, the proposed method EBWO-ResNet is feasible and can be used as an auxiliary method for maize disease identification. In the future, we will further explore to make up for the limitations of this experiment, for example, the dataset of this experiment is only from an open source platform, and we can try to collect datasets from more platforms next time, and even try to build experimental fields and collect datasets by ourselves. In this experiment, an improved method is used to optimize the BWO algorithm, and more improved mechanisms can be tried next time.There are many kinds of crops, and the follow-up work can also try to apply the method proposed in this paper to the identification of other crops.
